# Go With the Flow: Environmental Conditions Are Associated With Congruence Between eDNA Signals and Fish Communities in Small Lotic Ecosystems

**DOI:** 10.1002/ece3.74667

**Published:** 2026-11-15

**Authors:** Gerardo Petrosino, Lorenzo Talarico, Anna Rita Rossi, Lorenzo Tancioni, Eleonora Ciccotti, Chiara Leone, Paolo Franchini

**Affiliations:** ^1^ Department of Ecology and Biology (DEB) Tuscia University Viterbo Italy; ^2^ Department of Biology and Biotechnology “C. Darwin” Sapienza University of Rome Rome Italy; ^3^ Laboratory of Experimental Ecology and Aquaculture, Department of Biology University of Rome Tor Vergata Rome Italy

**Keywords:** 12S ribosomal DNA, electrofishing, environmental DNA, freshwater fish, invasive species, Italy, metabarcoding, quantitative estimation

## Abstract

Environmental DNA (eDNA) metabarcoding is increasingly used to assess fish communities. However, how environmental and hydrological conditions are associated with the eDNA signal, and how it relates to local fish assemblages, remains poorly understood. Here, we investigated whether environmental drivers modulate the spatial congruence between eDNA signals and local fish assemblages by comparing qualitative and quantitative fish data derived from eDNA metabarcoding and electrofishing surveys at 17 sites across eight watercourses in central Italy. Expectedly, metabarcoding detected more species than electrofishing at most sampling sites, reflecting higher sensitivity to rare taxa. eDNA read counts showed a moderate relationship with both fish abundance and biomass, with stronger associations for the latter. However, the congruence between eDNA and electrofishing‐derived community estimates varied along environmental gradients: while increased water discharge correlated with higher divergence between methods, temperature and oxygen showed the opposite trend. These results suggest that eDNA signals integrate both local and upstream biodiversity depending on hydrological and physicochemical conditions. Our findings support the high detection power of eDNA metabarcoding for characterizing fish communities in small rivers and suggest that the eDNA signal may integrate information from beyond the electrofished reach. More broadly, our study demonstrates that eDNA should be interpreted as an environmentally mediated signal rather than a direct proxy of local community composition. By linking variation in eDNA–community congruence to environmental gradients, this study provides a general framework for predicting when and where eDNA reflects the sampling area versus upstream biodiversity in flowing ecosystems.

## Graphical abstract

In this study, we investigated how environmental and hydrological drivers—such as water discharge, pH, temperature, and dissolved oxygen—are associated with the ability of the eDNA signal to qualitatively and quantitatively reflect the local fish community across streams. We found that the concordance between eDNA metabarcoding and electrofishing surveys varied along physicochemical gradients, with factors like water discharge increasing divergence by integrating upstream signals. Consequently, eDNA metabarcoding should be interpreted as an environmentally mediated signal rather than a direct proxy of local community composition.

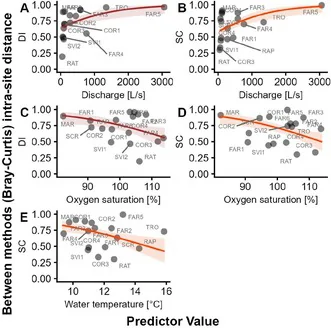

## 1 Introduction

Freshwater ecosystems host a disproportionate share of global biodiversity despite covering a small fraction of the Earth's surface, a pattern particularly evident in fish diversity, often referred to as the “freshwater fish paradox” (Tedesco et al. 2017). However, they are among the most threatened ecosystems worldwide due to anthropogenic pressures and climate change (Prakash 2021). Monitoring fish communities in these systems is therefore essential for understanding and mitigating biodiversity loss (Butchart et al. 2010; Turak et al. 2017).

Traditional approaches, such as electrofishing or fishery‐based monitoring, have long been used to assess fish assemblages, but they can be invasive, labor‐intensive, costly, and subject to detection biases (Nielsen 1998). Additionally, fish sampling requires specific expertise for both collection and taxonomic identification, and phenotypic plasticity, cryptic species, or challenges posed by the morphological characteristics of different life cycle stages can lead to uncertainties and errors that frequently require barcoding analysis for accurate identification (Bingpeng et al. 2018; Kirsch et al. 2018; Xing et al. 2022).

In the last decades, metabarcoding of environmental DNA (eDNA)—i.e., DNA molecules shed by organisms into environmental matrices—has emerged as a powerful and noninvasive approach for biodiversity monitoring in aquatic systems, enabling the detection of multiple species from water samples and proving particularly effective for the assessment of fish diversity (e.g., Littlefair et al. 2021; Pont et al. 2021; Wang et al. 2021; Keck et al. 2022). For instance, metabarcoding appears to detect more taxa than bottom trawl surveys (Maiello et al. 2024; Zhang et al. 2025), and species presence is not significantly biased by the type of bioinformatic reference pipeline (dos Santos and Blabolil 2025). In addition, due to its high sensitivity, the eDNA approach is especially valuable for detecting rare, elusive, or invasive taxa. For the latter, eDNA metabarcoding can serve as the basis of an early warning system and for verifying the eradication of aquatic invasive species (AIS) (Keskin 2014; Davison et al. 2017), which can dramatically impact native biodiversity (Gozlan 2010; Cucherousset and Olden 2011).

Beyond its analytical advantages, eDNA represents a complex ecological signal shaped by the interplay of organismal, environmental, and hydrological processes. In flowing systems, eDNA amount is primarily a balance among continuous production, unidirectional transport, sediment‐like particle dynamics (settling, retention, and resuspension), and degradation, such that the signal detected at a given location may integrate biodiversity information across multiple spatial scales rather than reflecting only the local community (Harrison et al. 2019; Van Driessche et al. 2023). Importantly, experiments in controlled and natural systems indicate that key environmental variables—including discharge (Shogren et al. 2017; Van Driessche et al. 2023), temperature (Strickler et al. 2015; Tsuji et al. 2017; Jo et al. 2019), dissolved oxygen (Weltz et al. 2017; Huang et al. 2022), and pH (Strickler et al. 2015; Seymour et al. 2018; Yang et al. 2025)—regulate both the persistence and spatial distribution of eDNA. Consequently, these factors can alter the degree of correspondence between eDNA‐derived signals and locally sampled communities, although a clear consensus has yet to emerge.

Finally, although previous studies have reported a general congruence between eDNA‐based and traditional surveys of fish communities, the strength of this relationship is highly context‐dependent (Keck et al. 2022), making its ecological interpretation challenging. In particular, some aspects related to the sensitivity of eDNA‐based results are still not completely understood (Yao et al. 2022; Rishan et al. 2023, 2025; Liu et al. 2025), and reliable quantitative estimates of species richness are still difficult to achieve (Lacoursière‐Roussel et al. 2016; Fukaya et al. 2022; Losa et al. 2025), so that the combination of traditional and molecular approaches is still necessary to obtain accurate estimates (Curto et al. 2025; Liu et al. 2025).

Consequently, key unresolved questions include: (1) Whether eDNA provides a reliable proxy of local community composition or a spatially integrated signal influenced by upstream biodiversity, and (2) the extent to which eDNA read counts reflect species abundance or biomass, considering that eDNA quantity is likely influenced by multiple factors such as species‐specific shedding rates, environmental conditions, and hydrological transport processes. Disentangling these still poorly understood processes is critical for interpreting eDNA data in an ecological context, particularly in small lotic systems where hydrological variability and habitat heterogeneity may strongly modulate eDNA dynamics.

Italian freshwater ecosystems host a rich fish fauna, consisting of numerous endemic and subendemic species, often characterized by discontinuous and/or patchy distributions across the Italian peninsula (Lorenzoni and Carosi 2026). They co‐occur with a large number of non‐native and translocated taxa, whose presence, along with other anthropogenic pressures, is directly impacting the conservation status of Italian native freshwater fishes (Lorenzoni et al. 2021; Lorenzoni and Carosi 2026). Indeed, 23 species are currently included in the IUCN (International Union for Conservation of Nature) Italian Red List as threatened (in RE, CR, EN, and VU categories) (Rondinini et al. 2022). Moreover, in Mediterranean streams fish diversity is structured along marked environmental gradients across relatively small spatial scales, with distinct fish assemblages occurring along longitudinal river profiles (Lorenzoni et al. 2006; Oliveira et al. 2012; Grapci‐Kotori et al. 2020). These characteristics make Italian streams a useful model system to investigate how ecological and hydrological processes are associated with the eDNA signal and its correspondence with the local fish community.

To address these issues, we combined qualitative and quantitative data from eDNA metabarcoding and traditional electrofishing surveys across small streams in central Italy (Latium region). We aimed to evaluate the spatial congruence between fish assemblages retrieved by the two monitoring approaches and to investigate how local environmental drivers influence their relationship. Specifically, we: (1) assessed whether eDNA read counts reliably reflect quantitative population metrics, exploring their alignment with both fish absolute abundance and total biomass; and (2) determined how fluctuating hydrological and physicochemical conditions—primarily water discharge—modulate the consistency between methods. By explicitly linking eDNA signals to underlying ecological processes, this study provides a general framework for interpreting when and where eDNA reflects local versus spatially integrated biodiversity in flowing ecosystems.

## 2 Materials and Methods

### 2.1 Sampling Design and Procedures

Environmental DNA samples were collected in 17 sites in Central Italy (Latium Region) from 8 watercourses in 4 basins, representative of various freshwater environments: from Apennines upper course streams, where salmonids are dominant, to medium‐low altitude course inhabited by salmonids and cyprinids, and lowland cold small rivers, where salmonids, cyprinids and euryhaline fishes coexist (Figure 1 and Table S1) (Huet 1959; Gandolfi et al. 1991). Notably, to minimize disturbance to the reproduction and spawning of locally dominant taxa, we performed sampling during a 29‐day winter window in cyprinid‐dominated sites, while salmonid‐dominated sites were surveyed during a 9‐day summer window (i.e., before and after reproduction, respectively; see exact dates in Table S1).

**FIGURE 1:**
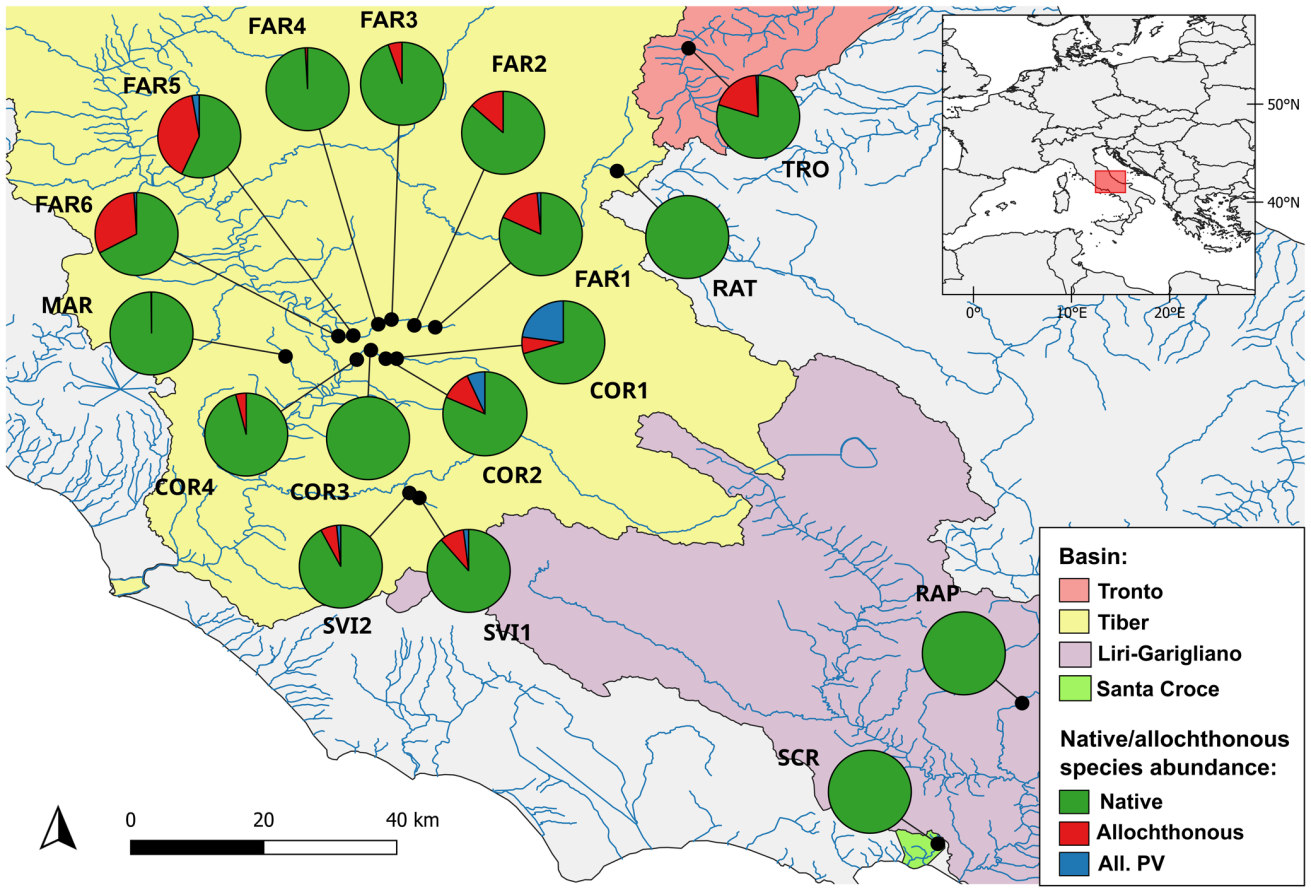
Geographic location of the study area (black and red boxes, top right) and distributions of sampling sites. Relative abundances of eDNA reads are shown for the sum of species native to the study area (Native), introduced from outside Italy (Allochthonous), or originating and introduced from the Padany‐Venetian ichthyogeographic district of northern Italy (All. PV), using different colors. Site abbreviations are reported in Table S1.

eDNA was collected by filtering two replicates of 2 L of water at each site (collected about 20 m apart) directly from the watercourse, using a vacuum pump (Smith‐Root, www.smith‐root.com/edna/edna‐citizen‐scientist‐sampler) and 0.45 μm pore size filter packs designed for eDNA sampling (Thomas et al. 2019). Filters were placed into single sterilized plastic bags and stored at −20°C until eDNA extraction. During sampling, operators used surgical masks and nitrile gloves to minimize contamination, and the pump was washed with ~15% sodium hypochlorite solution between sampling sessions. To assess the level of contamination induced by the pump, we collected field blanks by filtering 1 drinking water sample immediately before each sampling session, and further processed them in the same way as regular samples.

After water filtration, we captured fish by electrofishing, following the two‐pass removal method (Moran 1951; Zippin 1956) over a stretch of approximately 20 times the river width. After visual sorting, we annotated the number of individuals and cumulative biomass per species. We contextually recorded (average triplicate measures) water temperature, electric conductivity, pH, and oxygen saturation with a HI98194 multiparametric probe (Hanna Instruments), and estimated the discharge through the velocity‐area method. Fish sampling was authorized by the Latium Regional Direction responsible for Hunting and Fishing activities (prot. G07347/2022) and by Directions of Protected areas (prot. 528/2024).

### 2.2 DNA Extraction

eDNA was extracted from the filters in a dedicated laboratory, thoroughly cleaned with 10% sodium hypochlorite before any operation and where no other DNA manipulation occurred (i.e., extraction and amplification of non‐eDNA samples). We extracted eDNA from half of the inner membrane in the filter packs using the Quick‐DNA Miniprep kit (Zymo Research) according to the manufacturer's protocol for animal tissues. The final products were eluted in 60 μL of Elution Buffer (Zymo Research) and stored at −20°C.

Only samples collected from the same watercourse were extracted on the same day to minimize cross‐contamination, and an extraction blank (only reagents) was also produced each day.

### 2.3 DNA Amplification, Library Preparation, and Sequencing

We selected the Tele02 primer pair for 12S mitochondrial metabarcoding, based on its high taxonomic resolution (87% species, 100% order) (Taberlet et al. 2018) and established efficacy in marine and freshwater studies (Cananzi et al. 2022; Maiello et al. 2024; Shen et al. 2024). Furthermore, the resulting 120–228 bp fragment aligns with the consensus that barcodes under 200 bp enhance PCR performance (Bylemans et al. 2018), despite occasional contrary evidence (Zhang et al. 2020). To enable demultiplexing, unique 8 bp tags were added to the 5′ end of both primers, with at least 3 bp mismatches between pairs (Table S2) to mitigate tag jumping (Schnell et al. 2015). Additionally, 6 degenerate bases (2–4 Ns preceding the tag) were included to increase sequence length diversity and improve sequencing accuracy (Cananzi et al. 2022). PCR amplification was carried out in a total volume of 20 μL using a TC9610 Multigene OptiMax Thermal Cycler, and performed in triplicate for each sample. Field and extraction blanks, and PCR negatives for each run were also amplified as controls. Reaction mix and thermal cycling condition parameters are detailed in Table S3. Amplification products were checked on a UV transilluminator (2% agarose gel electrophoresis) before pooling triplicate samples; no signal was observed in the controls.

Pooled amplicons were subsequently quantified using Qubit Flex Fluorometer (ThermoFisher) to normalize before mixing into a single 1.5 mL tube. DNA concentration and fragment size in the all‐amplicons pool were checked using the 4150 TapeStation System (Agilent). Purification was performed using the MagSi‐NGSPREP Plus magnetic beads (Magtivio) to remove nontarget DNA fragments. The purified product was finally sent to an external service (BGI Genomics, https://www.bgi.com/global) for sequencing 2 × 150 bp on the DNBSEQ‐G400 platform. To check for contaminations across field and laboratory steps, one control per type (i.e., amplicons from one field blank, one extraction blank, and one PCR negative) was sequenced too.

### 2.4 Bioinformatic Analyses and Taxonomic Assignment

Raw reads were demultiplexed and tags trimmed using Cutadapt v5.0 (Martin 2011). Bioinformatic processing was performed in QIIME 2 v2024.10.1 (Bolyen et al. 2019) using the DADA2 pipeline (Callahan et al. 2016) for denoising, dereplication, chimera removal, and paired‐end merging under default parameters.

Taxonomic assignment of amplicon sequence variants (ASVs) was performed via BLAST+ (Bokulich et al. 2018) against an improved Mitohelper database (Lim and Thompson 2021). The database was integrated with 12S sequences for four endemic Italian species ( *Barbus tyberinus* , *Padogobius nigricans* , *Sarmarutilus rubilio*, and *Squalius lucumonis*) retrieved from various online sources (Table S4); notably, *S. rubilio* 12S was *de novo* sequenced for this study (GenBank no. = PX842949‐51) from samples collected by Petrosino et al. (2022). Following the 1.23% maximum intraspecific divergence estimated for fish (Wang et al. 2023), we performed and combined two assignment runs based on different identity thresholds: 99% for primary identification and 98% for secondary assignment of sequences with no matches in the primary identification. ASVs assigned to higher ranks than species or remaining unassigned were manually validated against the NCBI GenBank database (Altschul et al. 1990). After the assignment, all further analyses were conducted in R v4.4.2 (R Core Team 2025). Contaminants were identified and removed via the MICRODECON package v1.0.2 (McKnight et al. 2019), using the *decon* function (prop.thresh = 0.05) with samples grouped by site replicates: ASVs present in negative/blank controls were adjusted in “true” samples or removed if their frequency was < 5% within the group. Finally, non‐freshwater fish sequences and ASVs below the 98% threshold were discarded prior to taxonomic condensation and downstream analysis.

### 2.5 Fish Quantitative Estimates

eDNA read counts for each taxon in all samples were square‐root transformed to mitigate total sequencing depth bias and improve comparability across samples and sites (Mariani et al. 2021; Maiello et al. 2024). Site replicates were then merged by averaging read counts per taxon, using the resulting values (hereafter referred to simply as transformed read counts) as a proxy for eDNA abundance (Hänfling et al. 2016; Skelton et al. 2023).

For electrofishing data, per‐site abundance and biomass of each taxon were estimated using the Moran‐Zippin method. These values were subsequently standardized into Density Index (DI = individuals/m^2^) and Standing Crop (SC = g/m^2^) to account for variations in sampled surface area.

### 2.6 Comparison Between Methods

We first build Venn diagrams using the VennDiagram package v1.7.3 (Chen and Boutros 2011) to visualize the overall taxa detected by each method across all sites, also distinguishing between native and allochthonous species according to the Italian freshwater fish checklist (AIIAD 2021).

To evaluate whether eDNA metabarcoding outperformed conventional monitoring in detecting allochthonous species, which are often rare at the investigating sampling sites and thus challenging to capture, we performed R‐based Fisher's exact test of independence, McNemar's test for discordance, and calculated the Cohen's agreement coefficient *κ* to compare the detection rates of native versus non‐native species.

Differences in species composition and abundance between sites per method were calculated as pairwise Bray–Curtis distances, using the *vegdist* function in the Vegan package v2.7–2 (Oksanen et al. 2025), based on the transformed read counts per species (eDNA) or DI and SC (electrofishing). Then, we measured the correlation between the resulting distance matrices (reads vs. DI and reads vs. SC) using Mantel tests.

To investigate the correspondence between methods, data (transformed read counts, DI, and SC) were normalized by the maximum value of each dataset, and merged into reads‐DI and reads‐SC matrices. Intra‐site (i.e., inter‐method) and inter‐site dissimilarities per each matrix were then visualized through nonmetric multidimensional scaling (NMDS) based on semiquantitative Bray–Curtis distances, using the *metaMDS* function in the vegan package (Curto et al. 2025). To assess the environmental drivers of community structure, we fitted the environmental variables onto the NMDS ordination plot, using the *envfit* function with 999 permutations to assess statistical significance. Overall differences between eDNA and electrofishing were statistically evaluated via PERMANOVA (*adonis2* function).

We tested whether environmental parameters affected the concordance between fish assemblages assessed by the two methods across sampling sites, under the hypothesis that eDNA persistence and detectability could be influenced by the ecological features of the watercourses. Following a conceptual approach similar to (Talarico et al. 2025), we fitted Generalized Linear Models (GLM)—with a Beta distribution and a “logit” link function through the glmmTMB package v. 1.1.13 (Brooks et al. 2017)—to test water electrical conductivity, discharge, pH, oxygen saturation, water temperature, elevation, and sampling season as predictors of the intra‐site Bray–Curtis distance (response) between eDNA and electrofishing‐derived communities, with the latter expressed as Density Index (DI, in GLM1) or Standing Crop (SC, in GLM2), separately. All continuous predictors were individually scaled to ensure comparability among estimated effects. Season, conductivity, and elevation were not significant predictors in either model, and their exclusion improved model fit (ΔAIC = 2.1 and 5.7 for GLM1 and GLM2, respectively); marginal *R* ^2^ remained almost unchanged for GLM2 (0.781 → 0.778) but decreased moderately for GLM1 (0.620 → 0.525), reflecting the modest explanatory contribution of the removed, nonsignificant predictors. Considering the low sample size, we thus retained the simpler models, in which AIC favored parsimony over the marginal gain in fit, with discharge, oxygen saturation, pH, and water temperature as the final predictors. Preliminary analyses ensured the absence of substantial correlation (Spearman |*r*| ranged between 0.02 and 0.53) and multicollinearity (Variance Inflation Factors < 3.93) among predictors, as assessed via the performance package v. 0.15.3 on the full models (Lüdecke et al. 2021). We used DHARMa v. 0.4.8 (Hartig et al. 2026) to verify model assumptions and for diagnostics, namely residuals distribution, data (over)dispersion, outliers, and deviations from linearity of simulated residuals. To account for the hierarchical structure of our data (sites within watercourses) we initially included watercourse as a random effect –however, since 5 out of the 8 sampled rivers were represented by a single site, random intercepts were not reliably distinguishable from an overfitting artifact. Therefore, nonindependence of sites within the same river was instead addressed by computing cluster‐robust standard errors (cluster = watercourse) with a finite‐sample (HC1) correction (Cameron and Miller 2015) via the sandwich v. 3.1–3 package (Zeileis et al. 2020), with significance assessed with a Wald *Z*‐test. Finally, partial effect plots for significant predictors were visualized on the original response scale using the *plot_model* function from the sjPlot package v. 2.9 (Lüdecke 2025).

### 2.7 Relationship Between eDNA Proxies and Fish Abundance and Biomass

We tested the association between quantitative estimates of fish amounts returned by eDNA and electrofishing. Specifically, we tested whether eDNA transformed read counts predicted fish abundance (DI) or biomass (SC) of a certain species at a given sampling site, across the whole dataset. As the response variables (DI or SC) were zero‐inflated–namely included a large amount of zeros, possibly indicating false negatives due to traditional sampling errors/limitations (Martin et al. 2005)–we fitted the following zero‐inflated Gamma Generalized Linear Mixed‐effects Models (GLMM) which contextually allowed to: (1) model zeros as binary outcomes using a zero‐inflation component with a Bernoulli distribution and a “logit” link function, handling the probability of observing a zero; (2) model positive values using a conditional component with a Gamma distribution and a “log” link function, handling the magnitude of nonzero observations; (3) account for nonindependence of data from certain sampling sites or species, for instance due to species‐specific differences in eDNA shedding rates (Thalinger et al. 2021), by including random intercepts for species and sites.

$$\text{GLMM1}=\mathrm{DI}∼\text{reads}+\left(1|\text{species}\right)+\left(1|\text{site}\right)$$

$$\text{GLMM2}=\mathrm{SC}∼\text{reads}+\left(1|\text{species}\right)+\left(1|\text{site}\right)$$

We removed co‐absences (i.e., species missing in both eDNA and electrofishing in a certain site), which would artificially improve the robustness of models (Russo et al. 2021). We used the *glmmTMB* function of the homonymous package v1.1.13 (Brooks et al. 2017; McGillycuddy et al. 2025) for model fitting, and sjPlot for visualization of predictions.

## 3 Results

### 3.1 Taxa Identification, Abundance, and Distribution

After demultiplex and denoising, and excluding the control samples, we obtained overall 17,815,438 reads (mean per sample = 523,983.5 ± 382,733.3 SD, Table S5D) and 1776 total ASVs.

Taxonomic assignment at a 99% identity threshold identified 655 fish ASVs, with an additional 250 ASVs recovered by lowering the threshold to 98%. The combined runs identified 40 fish species, eight of which were classified as strictly marine ( *Merluccius merluccius* , *Oblada melanura* , *Sardinella aurita* , *Sparus aurata* , *Trisopterus luscus* , *Xiphias gladius* ) or non‐native anadromous ( *Oncorhynchus keta* , *Salmo salar* ), collectively accounting for 0.05% of the total reads. Their detection in inland freshwaters could be due to contamination in the filtering system or during laboratory processing; however, their distribution does not follow any particular pattern, as they occurred sporadically without clustering in specific samples, watercourses, or extraction/amplification batches, and they are absent from the sequenced negative controls (Table S5D). Given that these taxa are commonly used for human consumption, we hypothesize their presence in freshwaters may be linked to food waste or greywater input (Pont et al. 2018; McColl‐Gausden et al. 2021). Because of their clear exogenous origin, we safely excluded them from downstream analyses. Among the remaining 32 fish species, 11 were native to the study area and 21 were allochthonous (5 of them being native to the Padany‐Venetian ichthyogeographic district in North Italy—PV) (Figure 2A). We also found three ASVs assigned to *Lampetra* sp. However, given that *L. planeri* and *L. fluviatilis* are indistinguishable using mitochondrial markers (De Cahsan et al. 2020), and that *L. fluviatilis* has not been recorded in the study area in recent decades (AIIAD 2021), these sequences were assigned to *L. planeri* . Unassigned sequences were 48.87% of the total number of reads: 56.60% mammals (26.64% *Homo sapiens* ), 18.71% birds, 1.03% amphibians, 8.61% fish with identity < 98%, 0.23% arthropods, 3.21% bacteria, 11.60% without match. Among the contaminant ASVs found by the decontamination procedure, 16 belonged to 6 freshwater fish species (2 *Squalius lucumonis*, 3 *S. squalus*, 2 *Telestes muticellus* , 1 *Padogobius bonelli* , 2 *P. nigricans* , 6 *Salmo trutta* ), and 8 of them were totally removed from all the samples (1 *S. lucumonis*, 1 *S. squalus*, 2 *P. nigricans* , 4 *S. trutta* ).

**FIGURE 2:**
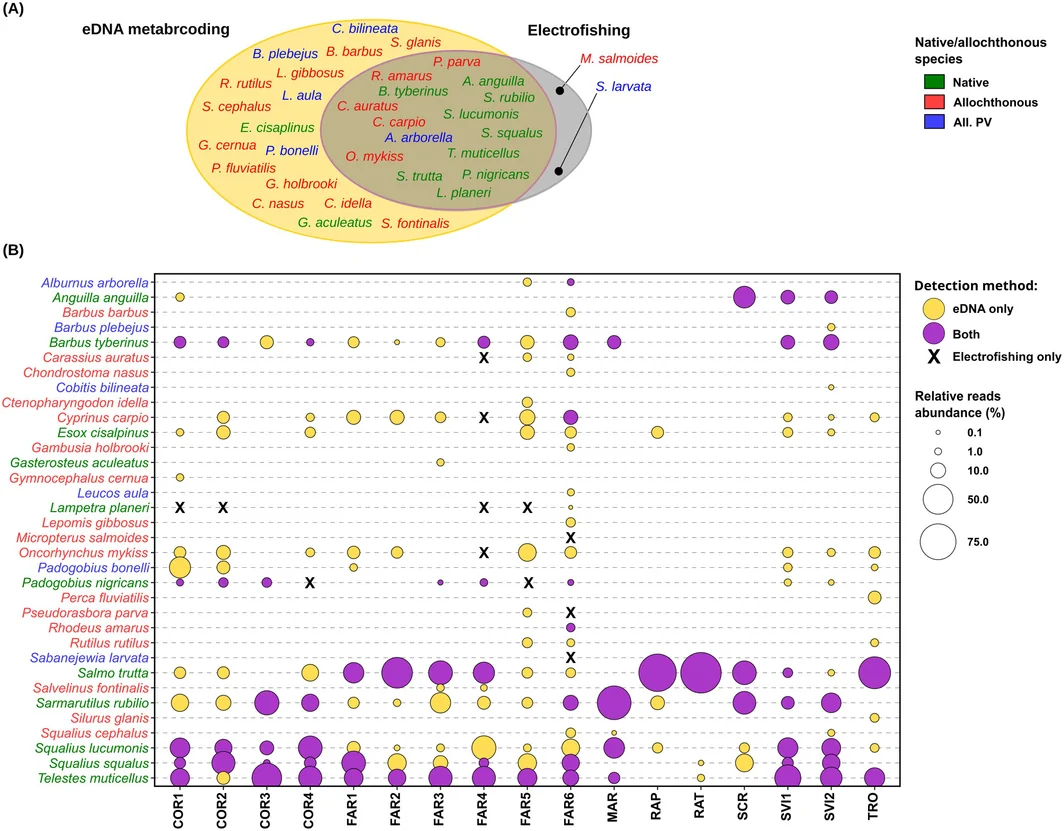
(A) Overall Venn diagram of the species detected by eDNA metabarcoding and/or electrofishing surveys; the size of the areas is proportional to the number of taxa. (B) Bubble plot of eDNA read counts; circle areas are proportional to the relative abundance of transformed eDNA reads for each taxon at each site; circle colors refer to the method by which the taxon was detected, with crosses indicating taxa detected by electrofishing only. For species text color, see the caption of Figure 1; site abbreviations refer to Table S1.

Electrofishing census identified 17 species (9 native and 8 allochthonous), all but two (the allochthonous *Micropterus salmoides* and *Sabanejewia larvata* ) also detected by eDNA (Figure 2A). Among the species found only by eDNA metabarcoding, we observed 15 allochthonous and only 2 native ones. The Fisher test revealed the number of allochthonous species detected by eDNA‐only was significantly higher than that found by traditional electrofishing (odds ratio = 7.88, *p*‐value < 0.05), with discordance between methods confirmed by the McNemar test (McNemar's *χ* ^2^ = 8.47, df = 1, *p*‐value < 0.01) and Cohen's *κ* (−0.18). Conversely, when repeating the analyses focusing on the number of native species, no statistical difference between methods was observed (Fisher and McNemar *p*‐value > 0.05; Cohen's *κ* = 0).

The total number of species detected per site across both methods ranged from 3 to 23. Based on eDNA data only, FAR5 and FAR6 exhibited the highest total richness (15 and 20 species, respectively) and allochthonous diversity (8 and 11 species). Conversely, RAP, RAT, and SCR showed low overall richness (3–5 species) and an absence of non‐native taxa (Figure 2B). Following read counts square‐root transformation and replicate merging, the five most frequent native taxa were *Salmo trutta* (21.52%), *Telestes muticellus* (19.99%), *Sarmarutilus rubilio* (12.68%), *Squalius lucumonis* (12.15%), and *Squalius squalus* (10.34%). The most frequent allochthonous species were *Oncorhynchus mykiss* (3.70%) and *Cyprinus carpio* (3.56%). Notably, *Salmo trutta* herein actually refers to the *S. trutta* species complex, which comprises Mediterranean‐native, exotic Atlantic‐farmed lineages, and their hybrids (Meraner and Gandolfi 2017). Considering that they are indistinguishable via the 12S marker, we categorized *S. trutta* as native for the purposes of this study. Relative frequencies per site (Figure 2B; Table S5C) identified *S. trutta* as the dominant native taxon in six sites, followed by *T. muticellus* (4 sites), *S. lucumonis* (3 sites), and *S. squalus* (2 sites). Only COR1 was dominated by an allochthonous species ( *Padogobius bonelli* ). The highest proportions of non‐native reads were recorded in FAR5 (39.61%), FAR6 (31.14%), and COR1 (29.40%), with all other sites remaining below 21% (Figure 1).

Electrofishing sampling yielded a species richness ranging from 11 (FAR6) to 1 (RAP and RAT, where only *S. trutta* was captured). Allochthonous taxa were restricted to FAR4 (3 species) and FAR6 (6 species). When pooled across all sampling sites, the highest cumulative DIs (Table S5A) were recorded for native *T. muticellus* (7.44, representing 40.17% of the total DI sum across all species and sites), *Padogobius nigricans* (5.68, 30.68%), *S. trutta* (1.75, 9.46%), and *S. lucumonis* (1.56, 8.44%). Among non‐native taxa, *Alburnus arborella* (0.09, 0.50%) and *Rhodeus amarus* (0.04, 0.19%) showed the highest DI. *S. trutta* was the most abundant (higher DI per site) taxon in 6 sites, followed by *P. nigricans* and *T. muticellus* (5 sites each), and *S. rubilio* (1 site). Biomass estimates (SC; Table S5B) partially mirrored eDNA reads frequencies, with the highest overall values observed for *S. trutta* (101.8, 50.15% of the total biomass), *T. muticellus* (33.34, 16.42%), *S. squalus* (14.62, 7.20%), *S. rubilio* (12, 5.91%), and *S. lucumonis* (10.24, 7.20%). For allochthonous species, the highest SC values were recorded for *C. carpio* (8.32, 4.10%) and *O. mykiss* (2.93, 1.45%). *S. trutta* showed the highest SC in 8 sites, followed by *P. nigricans* and *T. muticellus* (3 sites each), *S. rubilio* (2 sites), and *C. carpio* (1 site, FAR6).

### 3.2 Comparison Between Methods

eDNA metabarcoding detected more species than electrofishing in all sites except FAR4 (8 vs. 9). Almost the same species were found by both methods in COR3 and MAR, whereas the highest difference was observed in FAR5 (14 taxa found only by eDNA and 2 only by electrofishing) and FAR6 (12 taxa only by eDNA and 3 only by electrofishing) (Figure 2B). Fish assemblages retrieved by the two sampling methods moderately correlated (Mantel tests: *r* = 0.48, *p*‐value < 0.001 between reads and DI, and *r* = 0.56, *p*‐value < 0.001 between reads and SC) according to Bray Curtis pairwise distances (Table S6).

NMDS plot of distances for combined matrices revealed the eDNA convex hull was smaller than the electrofishing one for both DI and SC, and partially overlapping (Figure 3). For all methods, inter‐site differences were distributed mainly along the first axis, which showed the best correlation with elevation (Elev *r* ^2^ = 0.35 and 0.35 with *p*‐values < 0.01 for DI‐ and SC‐based plots, respectively) and water temperature (T *r* ^2^ = 0.36 and 0.32 with *p*‐values < 0.01 for DI‐ and SC‐based plots, respectively). Note that, while a negative correlation between these two variables is typically expected, their synchronous direction in this study likely results from the sampling seasonality, as high‐altitude sites were mostly sampled in summer and lower‐altitude sites in winter (Table S1). Conversely, no significance was observed for the remaining environmental variables. Intra‐site (between methods) differences (DI‐based mean = 0.72 ± 0.22, SC‐based mean = 0.67 ± 0.21) occurred mainly along the second axis. Highest intra‐site distances for eDNA vs. DI were observed in FAR5, TRO, FAR2, FAR3, and the lowest in RAT. For eDNA vs. SC the highest distances were observed in FAR5, MAR, COR1, FAR3, COR2, and the lowest in RAT and COR3 (Figure S1). The PERMANOVAs indicated a significant difference between methods, either when testing eDNA vs. DI (*F*_1,32_ = 5.69, *p* = 0.001) or eDNA vs. SC (*F*_1,32_ = 4.39, *p* = 0.001), explaining the 15.11% and the 12.06% of the total variance, respectively.

**FIGURE 3:**
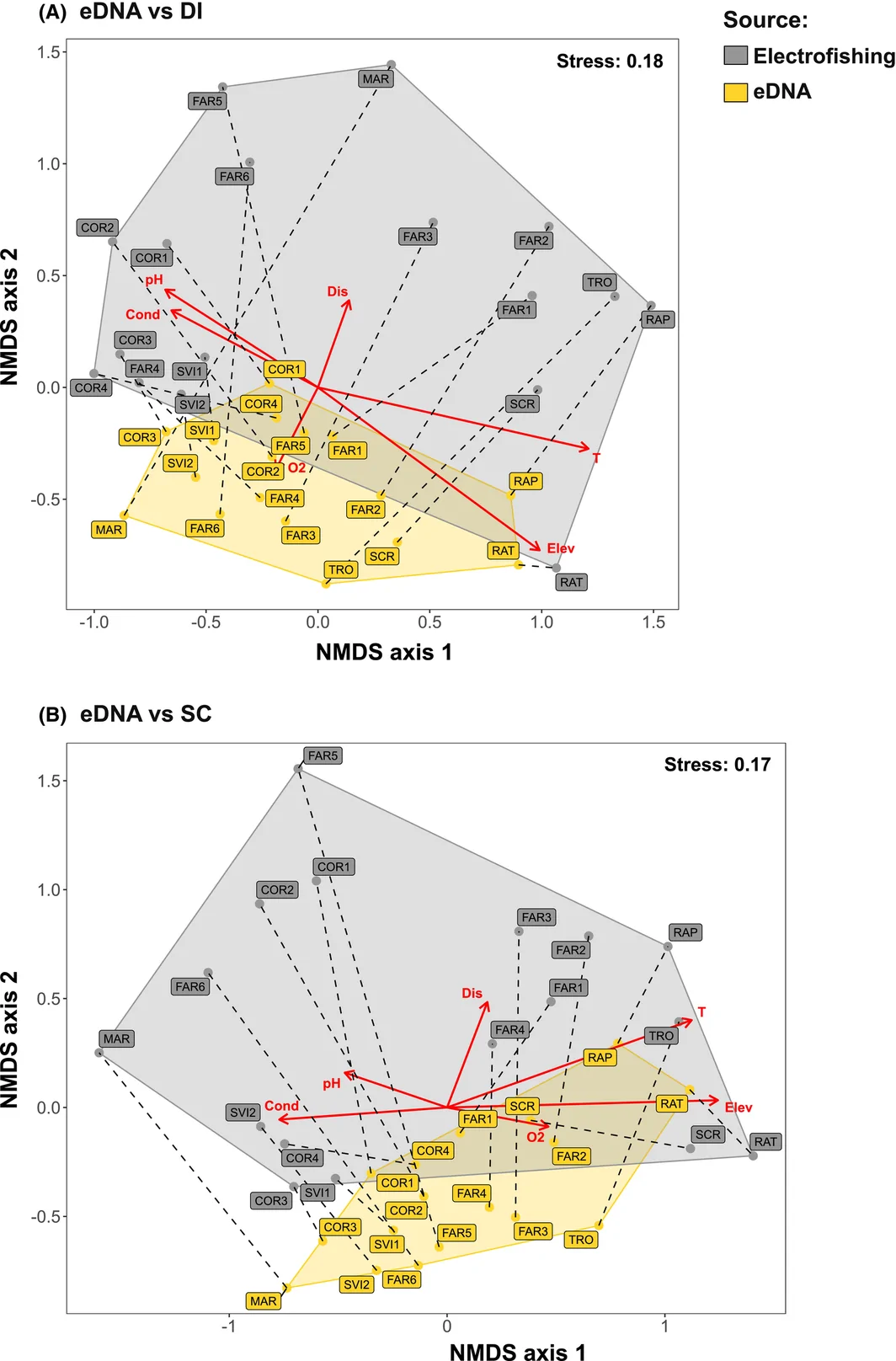
NMDS plot of Bray–Curtis distances among sites based on eDNA read counts and estimates of DI (A) and SC (B) from electrofishing surveys. Dotted segments join observations from the same sampling sites. Site abbreviations refer to Table S1. Red arrows represent the direction of fitted environmental variables: Elevation (Elev), water temperature (T), electric conductivity (Cond), pH, and oxygen saturation (O_2_).

Fitted GLMs indicated that the discordance between fish assemblages inferred by the two methods was significantly influenced by some environmental drivers (Table 1, Figure 4). Specifically, discharge and oxygen saturation showed the same direction and significance in both models (positive and negative, respectively), whereas water temperature was significant only for SC (GLM2). pH, sampling season, conductivity, and elevation were not significant predictors in either model (Table 1). The two models explained a relatively high variance (*R* ^2^ = 52.5% and 77.8% for GLM1 and GLM2, respectively). Model diagnostics confirmed that no model assumptions were violated (*p* > 0.05 for all DHARMa tests).

**TABLE 1:** Generalized linear models (GLM) with a beta error distribution family and a logit link function, assessing the relationship between Bray–Curtis intra‐site distance and four (scaled) environmental predictors—selected after excluding season, conductivity, and elevation (nonsignificant in both models; see Methods)—for 17 sampling sites.

Predictors	GLM1 (Bray–Curtis distance with DI)			GLM2 (Bray–Curtis distance with SC)		
	Estimates (95% CIs)	*Z* statistic	*p*	Estimates (95% CIs)	*Z* statistic	*p*
(Intercept)	1.07 (0.53 to 1.62)	3.85	**< 0.001**	0.86 (0.62 to 1.10)	7.03	**< 0.001**
Discharge	0.76 (0.19 to 1.33)	2.59	**0.010**	0.81 (0.54 to 1.09)	5.80	**< 0.001**
Oxygen saturation	−0.51 (−0.93 to −0.09)	−2.36	**0.018**	−0.53 (−1.02 to −0.05)	−2.18	**0.030**
pH	0.42 (−0.15 to 0.98)	1.45	0.148	0.43 (−0.20 to 1.06)	1.34	0.180
Water temperature	0.05 (−0.27 to 0.38)	0.32	0.747	−0.47 (−0.59 to −0.34)	−7.10	**< 0.001**
Observations	17			17		
*R* ^2^	0.525			0.778		

*Note:* Intra‐site distances refer to semiquantitative differences between communities retrieved by eDNA and electrofishing, considering fish density (DI = density index; GLM1) or fish biomass (SC = standing crop; GLM2). Estimates, 95% confidence intervals, *Z* statistics, and *p*‐values (statistically significant values are in bold) are based on cluster‐robust standard errors (HC1 correction; cluster = watercourse) to account for the nonindependence of sites sampled within the same river.

**FIGURE 4:**
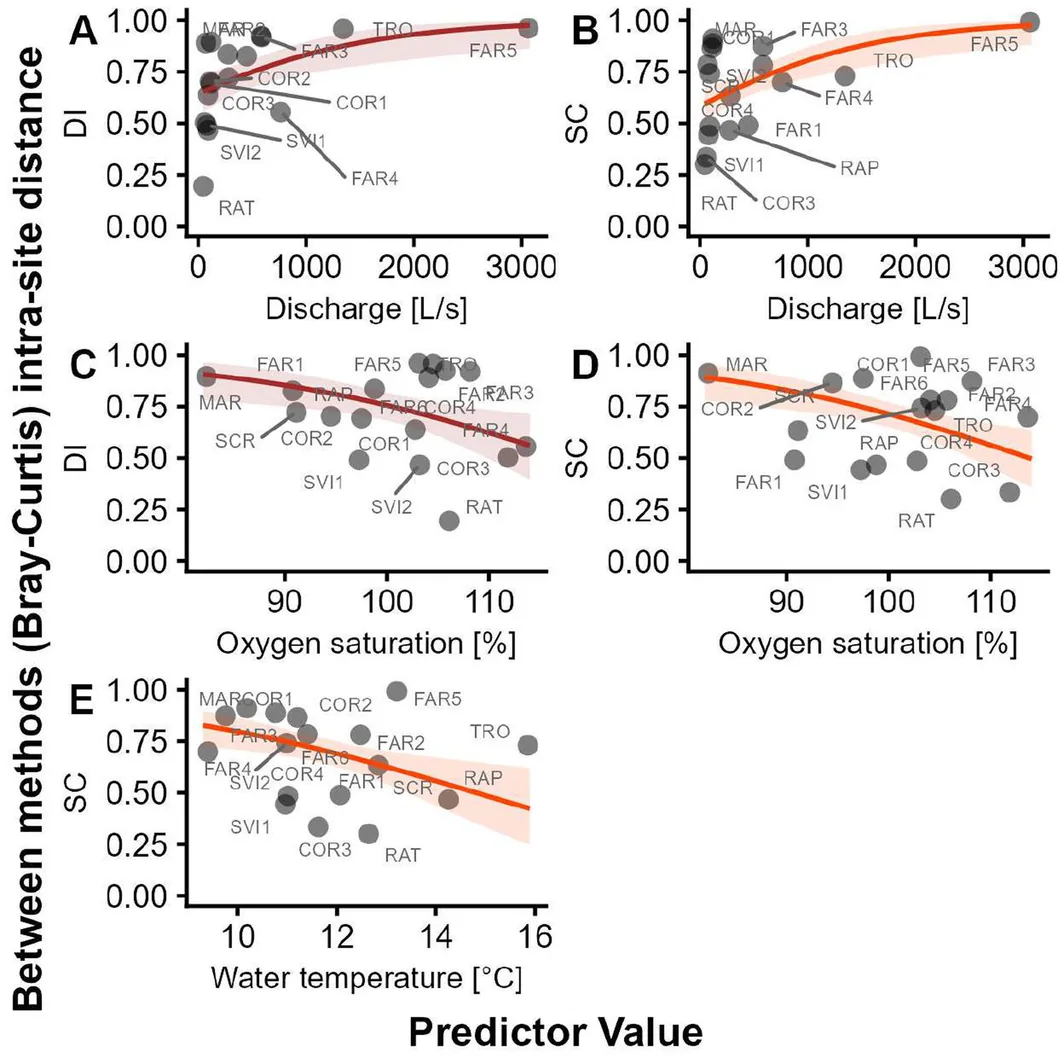
Partial effect plots depicting significant relationships (with 95% confidence intervals, shaded areas) between intra‐site Bray–Curtis distance and various environmental predictors, as obtained by fitted GLMs (Table 1). Bray–Curtis distances were calculated using fish density (DI, panels A and C) or biomass (SC, panels B, D, and E).

Zero‐inflated Gamma GLMMs revealed that the number of eDNA reads was a highly significant positive predictor for both DI and SC (*p* < 0.001; Figure S2). While fixed effects accounted for a modest portion of the variance (marginal *R* ^2^ = 0.171 and 0.225 for DI and SC, respectively), the models achieved high overall explanatory power when incorporating species and site as random effects (conditional *R* ^2^ = 0.866 for DI and 0.646 for SC; Table 2).

**TABLE 2:** Gamma generalized linear mixed‐effects models (GLMM) evaluating the effects eDNA read abundance (number of reads) on fish density and biomass (expressed as DI or SC in GLMM1 and GLMM2, respectively), while accounting for species and site membership (random effects).

Predictors	Response = Density index (GLMM1)			Response = Standing crop (GLMM2)		
	Estimates (95% CIs)	*Z* statistic	*p*	Estimates (95% CIs)	*Z* statistic	*p*
Conditional model (Gamma)						
(Intercept)	−4.041 (−5.015 to −3.067)	−8.131	**< 0.001**	−0.670 (−1.474 to 0.134)	−1.633	0.102
eDNA reads	0.007 (0.004 to 0.009)	5.337	**< 0.001**	0.006 (0.003 to 0.009)	3.877	**< 0.001**
Zero‐inflated model						
(Intercept)	1.474 (0.520 to 2.429)	3.027	**0.002**	1.474 (0.520 to 2.429)	3.027	**0.002**
eDNA reads	−0.011 (−0.017 to −0.005)	−3.847	**< 0.001**	−0.011 (−0.017 to −0.005)	−3.847	**< 0.001**
Random Effects						
*σ* ^2^	0.54			0.88		
*τ*_00_	1.33_site_			0.48_site_		
	1.45_species_			0.57_species_		
*N*	17_site_			17_site_		
	34_species_			34_species_		
Observations	172			172		
Marginal *R* ^2^/Conditional *R* ^2^	0.171/0.866			0.225/0.646		

*Note:* Coefficients are presented separately for the conditional (magnitude) and zero‐inflated (probability of zero) parts: Estimates with 95% confidence intervals of each predictor, along with *p*‐values from *Z*‐tests (statistically significant values are in bold). The residual variance (*σ* ^2^) and the between‐group variance (*τ*_00_) of random effects are shown with their sample size (*N*). Marginal and conditional *R* ^2^ indicate the proportion of variance explained by fixed effects (i.e., by combining conditional and zero‐inflation model parts) and by the whole model (i.e., fixed effects plus random effects).

## 4 Discussion

In this study, we found that eDNA and electrofishing samplings returned moderately congruent fish assemblage profiles, both qualitatively and quantitatively, even within small Mediterranean watercourses. Importantly, the observed discrepancies between these methods were strongly structured by environmental conditions, reflecting the underlying ecological and hydrological processes governing eDNA production, transport, and degradation rather than inherent methodological noise. Collectively, these findings underscore that eDNA metabarcoding is an environmentally mediated signal that integrates fish biodiversity across broader spatial scales, rather than a direct proxy of local community composition. Consequently, it should be viewed as a powerful complementary tool to traditional surveys, particularly effective for assessing the fish community at the watercourse scale rather than as a direct replacement for site‐specific electrofishing.

Regarding the performance of our metabarcoding protocol, we found that 49% of the ASVs obtained belonged to other vertebrates. This is not surprising, as comparative analyses of primers used in eDNA‐metabarcoding have shown that those targeting teleost fishes can also amplify sequences from other vertebrates (Zhang et al. 2020). Despite this limitation, there is a general consensus on using 12S primers in fish eDNA studies (Blackman et al. 2024), as they outperform those targeting other mtDNA genes in both amplification efficiency and biodiversity detection (Zhang et al. 2020).

Our metabarcoding approach identified more taxa than electrofishing at nearly all sampling sites. This result aligns with the growing consensus that eDNA‐based assessment often outperforms traditional survey methods in detecting overall species diversity (Fujii et al. 2019; Zou et al. 2020; Chen et al. 2025; Lin et al. 2025; Mlinarec et al. 2025). However, this superior detection power does not imply a total decoupling of methods; indeed, meta‐analyses have demonstrated a broad correspondence between freshwater fish community profiles derived from both molecular and conventional surveys (Keck et al. 2022).

Methodological discrepancies, when present, are primarily scale‐dependent. In larger watercourses, eDNA transport distance and the spatial coverage of traditional sampling often operate at different spatial scales and accessibility constraints may limit traditional surveys, leading to a potential underestimation of biodiversity compared to eDNA (Pont et al. 2021; Astorg et al. 2025; Curto et al. 2025). Therefore, in the small streams surveyed in this study, which were almost entirely wadeable, we expected a higher concordance in species detection between methods. Nevertheless, electrofishing‐based community assessments may fail to detect elusive or highly vagile species (Kanno et al. 2009; Pottier et al. 2020). Statistical analyses (Fisher's exact test, McNemar's test and Cohen's *κ*) revealed that eDNA metabarcoding detected more allochthonous taxa than electrofishing. Previous sampling using traditional methods (electrofishing) in the same watercourses investigated by this study reported these species as rare (Sarrocco et al. 2012), so they may have been missed during our sampling. Moreover, some allochthonous species could have been easily misidentified due to morphological similarities with native taxa, such as *Barbus barbus* and *B. plebejus* vs. *B. tyberinus* , *Leucos aula* and *Rutilus rutilus* vs. *Sarmarutilus rubilio*, and *Squalius cephalus* vs. *S. squalus*. This is consistent with evidence that eDNA metabarcoding is highly sensitive even at low population densities (Sandré et al. 2025) and is suitable for detecting cryptic (Carvalho 2025) rare or elusive species that may remain undetected by electrofishing (Fediajevaite et al. 2021; Shelton et al. 2019). However, in some cases, eDNA metabarcoding alone may not be able to capture the overall diversity due to low genetic divergence between closely related species, ecotypes, and Evolutionarily Significant Units within the same taxon (e.g., the *Lampetra* and *Salmo* genera aforementioned in the Materials and Methods section), whose distinction requires more in‐depth analysis.

Most of the allochthonous taxa identified exclusively by eDNA metabarcoding were predominantly localized within the lower reaches of the Farfa River (sites FAR5 and FAR6). These sites are located 3.0–6.5 km upstream of a reservoir on the Tiber River mainstream, where these taxa are well established (Sarrocco et al. 2012), suggesting an ongoing upstream expansion process (Lin et al. 2025). In our study, therefore, the most informative result was not the greater number of species detected by eDNA per se, but the taxonomic identity of the additional detections. Remarkably, most taxa uniquely recovered through metabarcoding were non‐native species occurring at apparently low abundance. These findings reinforce the greater sensitivity of eDNA metabarcoding for rare taxa, and thus its efficacy in characterizing invasion dynamics at the catchment scale, by revealing populations that remain below the detection threshold of traditional electrofishing surveys.

In some cases, eDNA can produce false negatives, for example due to inefficient PCR amplification or incomplete reference databases (Beng and Corlett 2020). For instance, the brook lamprey ( *Lampetra planeri* ) was captured in four sites, but detected by eDNA in only one. This discrepancy may be explained by the low amplification efficiency of the 12S marker for this taxon. Indeed, previous studies have shown that the 12S marker may fail to detect lamprey species, in contrast to the 16S marker (Gehri et al. 2021), or may exhibit reduced amplification efficiency (Macher et al. 2021). Additional occurrences detected exclusively by electrofishing were observed in the lower section of the Farfa River, where some of the highest values of water discharge were recorded. This pattern may be explained by complex plume dynamics affecting eDNA transport, which can lead to very low concentration at distance of 300–1000 m downstream from the source, as demonstrated in experiments using caged fishes in natural river systems (Van Driessche et al. 2023).

When considering quantitative data, we observed two main patterns in the spatial ordination of samples. Overall, eDNA and electrofishing captured broadly similar ecological gradients across sites. Indeed, inter‐site differences for both methods (metabarcoding and electrofishing) were primarily aligned along the X‐axis of the ordination plot and correlated with elevation. As expected based on the known distribution of fish communities in Europe (Huet 1959; Illies and Botosaneanu 1963; Gandolfi et al. 1991), this gradient clearly separates high‐elevation sites dominated by salmonids (e.g., RAP, RAT, TRO), mid‐elevation sites, characterized by mixed assemblages of salmonids and cyprinids (FAR1‐4 sites), and lower‐elevation sites dominated by cyprinids (COR, SVI, MAR, FAR5, and FAR6). The fact that both methods recovered the same longitudinal ecological gradient suggests that eDNA retained the “core” information on community composition across the river network. Conversely, intra‐site differences were generally aligned along the second axis, and PERMANOVA results supported a significant divergence between communities estimated by the two methods. However, Mantel tests and the GLMMs indicated a moderate correlation between them. Together, these results suggest that eDNA and electrofishing provide partially congruent views of fish assemblages: while both approaches describe the same large‐scale patterns of community turnover, eDNA does not necessarily reproduce the exact local abundance structure measured by electrofishing.

Read counts and eDNA concentrations represent an emergent ecological signal shaped by the combined effects of organismal biomass, species‐specific shedding rates, and environmental variables; consequently, whether they can be considered reliable proxies for fish biomass or abundance remains a matter of debate. In a literature review (Rourke et al. 2022) nearly 90% of the surveyed studies reported some form of relationship with quantitative biological data. While direct relationship has been reported particularly for fish in closed or semi‐closed systems, this link has proven to be inconsistent or only partially reliable in other environmental contexts (Rourke et al. 2022; Blackman et al. 2024), especially in large lakes and marine environments (Yao et al. 2022). Moreover, PCR biases in multispecies assessment (i.e., metabarcoding) may further complicate the relationship between eDNA read counts and biomass or abundance (Shu et al. 2021; Yao et al. 2022).

Both Mantel correlations and GLMMs consistently indicated that eDNA read abundance tracked fish biomass more closely than fish density. This pattern is mechanistically plausible because DNA shedding is expected to scale more strongly with organismal mass than with the number of individuals alone, and it's in accordance with several other studies (Maruyama et al. 2014; Taberlet et al. 2018; Yates et al. 2021; Astorg et al. 2025). Furthermore, the substantial variance explained by random effects in the GLMMs suggests that the relationship between eDNA concentration and fish metrics is strongly species‐specific and influenced by local environmental conditions (i.e., site‐dependent). Indeed, variation in eDNA shedding may occur among species (Andruszkiewicz Allan et al. 2021), due to differences in mucus production, epithelial and scale characteristics, swimming activity, and metabolic rate (Caza‐Allard et al. 2022, and references therein), as well as among age classes or individuals within the same species (Kelly et al. 2014; Talarico et al. 2026), and under different environmental conditions (Rourke et al. 2022). Consequently, eDNA read abundance should not be interpreted as a direct proxy of local biomass.

In the current study, we tested several environmental parameters against the semiquantitative (Bray–Curtis) intra‐site distances, and found that stream hydrology and spatial scale contribute to the divergence between electrofishing and eDNA‐derived community estimates. Notably, our GLMs showed that SC‐based distances were more strongly affected by these drivers than DI‐based ones, further supporting a tighter relationship between eDNA and fish biomass than abundance. To interpret how these parameters may influence discrepancies between methods, it is important to consider that eDNA concentration at a given site reflects a dynamic balance between local production and upstream inputs, and losses due to downstream transport and degradation (Rourke et al. 2022). Consequently, lower intra‐site distances (indicating higher methodological congruence) are expected when the eDNA signal mainly originates from the local fish community. Conversely, higher distances may result from substantial upstream inputs or prolonged eDNA persistence, leading to the detection of nonlocal and inflated eDNA quantities relative to a local biomass.

A central finding of this study is that the spatial scale represented by eDNA is not fixed, but varies predictably across environmental gradients. Under conditions that favor local signal retention, eDNA‐derived assemblages were generally more similar to locally sampled fish communities. Conversely, as the influence of transport processes increased, congruence progressively declined, suggesting a greater contribution of upstream biodiversity to the detected molecular signal. These findings reinforce the conceptualization of the riverine eDNA as an environmentally mediated and spatially integrated signal, rather than a direct, static proxy for local fish assemblage composition. Our interpretation is supported by the fact that water discharge showed the strongest positive correlation with intra‐site distance in our GLMs, likely reflecting increased transport of eDNA from upstream. According to (Pont et al. 2018), downstream transport is negligible in streams with discharge < 100 L/s, where the eDNA signal typically persists for less than one kilometer. Consistently, we observed the highest congruence between methods at sites below this threshold. Conversely, reaches characterized by higher discharge appear increasingly influenced by biodiversity signals originating upstream. The magnitude of this “noise” likely depends on local hydrology and cumulative upstream fish biomass (Van Driessche et al. 2023), and in our study it may explain the detection of unexpected taxa by metabarcoding. For example, the detection of game fish species, such as *Cyprinus carpio* , *Esox cisalpinus*, and *Oncorhynchus mykiss* , in small streams likely originates from recreational angling ponds connected to or located upstream of the sampled site (Blabolil et al. 2022). Similarly, in the mountainous TRO site, where a “simplified” salmonid‐dominated fish community was expected and high discharge was recorded, the eDNA metabarcoding instead reflected part of the community recorded in a 13 km upstream lake (i.e., Lake Scandarello), such as the wels catfish ( *Silurus glanis* ) and the perch ( *Perca fluviatilis* ) (Sarrocco et al. 2012). Moreover, the combination of downstream eDNA transport, species ecological traits and habitat heterogeneity may explain detection discrepancies involving native taxa ( *Barbus tyberinus* , *Sarmarutilus rubilio*, *Squalius lucumonis, Padogobius nigricans*), and the allochthonous *Padogobius bonelli* . These species have specific mesohabitat requirements and may be absent from the short reaches sampled by electrofishing if suitable habitats (i.e., riffles or pools), are lacking locally. However, eDNA can detect them by integrating signals from nearby habitat patches located only a few meters away (Pont et al. 2018; McColl‐Gausden et al. 2021). According to our results, disagreement between eDNA and electrofishing should not necessarily be interpreted as reduced accuracy of the molecular method, but rather as evidence that the two approaches integrate ecological information over different spatial extents.

The GLMs also revealed that methodological convergence in fish assemblage reconstruction increased with water temperature and oxygen saturation—although the former was significant only for the SC‐based comparisons. Both variables are known to influence eDNA decay rates. Higher temperatures and oxygen levels can promote denaturation, hydrolysis, and oxidation processes (Joseph et al. 2022 and references therein), as well as stimulate microbial activity responsible for eDNA degradation, as shown in microcosm experiments (Strickler et al. 2015; Tsuji et al. 2017; Jo et al. 2019; Caza‐Allard et al. 2022). However, in our study abiotic and microbial‐based degradation likely affect equally local and nonlocal eDNA. Additionally, higher values of temperature and oxygen level may enhance fish metabolic activity, increasing eDNA shedding by local communities (Stewart 2019), and also capture probabilities in traditional surveys (McInerny and Cross 2000), thereby improving concordance between methods. Finally, pH did not emerge as a significant predictor of intra‐site divergence between methods. This does not necessarily rule out a biological effect, which is documented under controlled conditions: accelerated eDNA degradation has been reported under acidic conditions (pH < 7.5), likely via increased depurination and hydrolysis (Strickler et al. 2015; Seymour et al. 2018; Yang et al. 2025), while peak decay rates have also been identified around pH 8, coinciding with the optimal enzymatic activity of microbial DNase (Jo 2026). Rather, unlike discharge and elevation—which respectively integrate hydrological conditions over the whole reach and represent a more stable geographic attribute of each site—pH (as well as conductivity) is a spot measurement that can fluctuate substantially over short temporal and spatial scales, potentially weakening the correspondence between a single‐point reading and the conditions actually experienced by eDNA along its transport pathway. Disentangling the relative roles of abiotic and microbial factors controlling eDNA persistence in lotic networks remains an important direction for future research. Sampling season, similarly, was not a significant predictor in either model, indicating that the temporal split in our sampling design did not itself substantially confound the observed environmental relationships. It is fair noticing that, because of the hierarchical structure of sampling (sites were nested within rivers, plus several rivers represented by a single site), the effective number of independent sampling units may be lower than 17—our cluster‐robust sensitivity analysis indicated that the associations reported above are generally robust to nonindependence of observations, but the small number of rivers should be considered when interpreting their magnitude. Beyond its theoretical implications, our research provides a basis for calibrating eDNA metabarcoding protocols to meet increasingly stringent freshwater biomonitoring mandates. By demonstrating higher detection rates for some rare or elusive taxa compared to traditional electrofishing, this study underscores the potential of molecular tools to enhance routine biodiversity assessment under current conservation directives (e.g., European Water Framework Directive EC 2000, and Habitats Directive EEC 1992). This higher detection rate is particularly relevant to the monitoring of rare or elusive species of conservation concern. Additionally, by detecting non‐native species at low read abundances, eDNA may contribute to early‐warning efforts for invasive species management, facilitating compliance with regulations on invasive alien species (e.g., EU 2014, 2017; dos Santos and Blabolil 2025). Furthermore, the moderate yet significant correlation between eDNA read counts and fish biomass provides a promising avenue for exploring the quantitative biotic indices relevant to these regulatory frameworks.

## 5 Conclusions

Our results show that eDNA metabarcoding provides a reliable representation of fish assemblages in small Mediterranean streams, while offering substantially greater sensitivity for the detection of rare and non‐native species compared to conventional survey. While eDNA read counts showed a moderate correlation with local fish biomass—an association stronger than that observed with individual abundance—this relationship was heavily modulated by environmental conditions. Crucially, the spatial scale represented by eDNA is inherently dynamic; hydrological and physicochemical variables determine the extent to which molecular signals reflect localized communities or integrate biodiversity information from upstream reaches. Consequently, discrepancies between eDNA and electrofishing should not be dismissed as methodological noise, but rather interpreted as evidence that these two approaches capture ecological information across distinct spatial scales.

By elucidating the environmental drivers that modulate eDNA‐community congruence, this study establishes a predictive framework for interpreting eDNA as an environmentally mediated signal in lotic ecosystems, facilitating its integration into high‐resolution biodiversity monitoring programmes.

## Author Contributions

**Gerardo Petrosino:** conceptualization (equal), formal analysis (equal), investigation (equal), methodology (equal), visualization (equal), writing – original draft (equal), writing – review and editing (equal). **Lorenzo Talarico:** conceptualization (equal), formal analysis (equal), investigation (equal), methodology (equal), visualization (equal), writing – original draft (equal), writing – review and editing (equal). **Anna Rita Rossi:** conceptualization (equal), funding acquisition (equal), supervision (equal), writing – original draft (equal), writing – review and editing (equal). **Lorenzo Tancioni:** conceptualization (equal), funding acquisition (equal), investigation (equal), methodology (equal), supervision (equal), writing – review and editing (equal). **Eleonora Ciccotti:** funding acquisition (equal), writing – review and editing (equal). **Chiara Leone:** writing – review and editing (equal). **Paolo Franchini:** funding acquisition (equal), supervision (equal), writing – review and editing (equal).

## Funding

This study was funded by the “Italian Ministry of University and Research” through the PRIN 2022‐2022JJ7STN project.

## Ethics Statement

Fish sampling procedures were in accordance with the relevant legislation (CEN EN 131 14011/2003‐Water quality‐Sampling of fish with electricity) and were authorized by Latium Regional Direction responsible for Hunting and Fishing activities (prot. G07347/2022) and by Directions of Protected areas (prot. 528/2024).

## Conflicts of Interest

The authors declare no conflicts of interest.

## Supporting information


**Figure S1:** Boxplots of Bray–Curtis intra‐site distances calculated using the number of eDNA reads (eDNA) and fish density (DI) or biomass (SC).
**Figure S2:** Associations between proxies of eDNA and fish density and biomass. Plots depict the relationships (with 95% confidence intervals; shaded area) between square‐root transformed eDNA reads and (A) fish abundance (DI) or (B) biomass (SC). Predictions are derived from the zero‐inflated Gamma GLMMs presented in Table 2.

**Table S1:** Details for the 17 sampling sites (Sites ID) from Central Italy: name of the basin and watercourse; sampling date as dd/mm/yyyy, and season; geographic coordinates (Datum WGS84); elevation (Elev.) as meters above mean sea level; water temperature (T) expressed in degrees Celsius; pH of the water (pH); oxygen saturation in water (O_2_) in percentage; water electric conductivity (Cond.) in microsiemens per cm; discharge (Dis.) expressed in liters per second. For watercourses with multiple sampling sites, the numeric suffix in the site ID reflects the spatial sequence from upstream (1) to downstream.
**Table S2:** Unique per sample nucleotide tags and degenerate bases (N) added to the 5′‐end of forward and reverse primers.
**Table S3:** PCR conditions (A) and reaction mix (B) applied for the amplification of the 12S marker from eDNA samples.
**Table S4:** List of 12S sequences for 4 taxa native of the investigated area, retrieved from online databases BOLD (https://v4.boldsystems.org/) and Genbank (https://www.ncbi.nlm.nih.gov/genbank/), and added to Mitohelper for taxonomic assignment. Sequences for Sarmarutilus rubilio were obtained in this study and then submitted to Genbank.
**Table S5:** Diversity Index (A) and Standing Crop (B) values per site calculated for the taxa (order; family; genus; species) identified by electrofishing procedures. Number of eDNA reads (C) after decontamination, non‐fish and marine‐fish reads removed, and square‐root normalization and sample merging per site. (D) Number of eDNA reads per replicate and negative controls after sequencing and taxa identification; NEG_FIELD, NEG_EXTR, and NEG_PCR indicate controls obtained during field sampling, eDNA extraction, and samples amplification, respectively. (E) Number of eDNA reads per replicate after decontamination. Site abbreviations refer to Table S1; replicates for the same site are indicated with A and B at the end of the site ID.
**Table S6:** Bray–Curtis distance matrices between pairs of sampling sites, based on eDNA number of reads (A) and electrofishing estimates (B) of DI (below the diagonal) and SC (above the diagonal). The background color gradient is related to the distance amount: white indicates the lowest values, while dark green indicates the highest ones.

## Data Availability

Raw sequencing data are available in the NCBI Sequence Read Archive (SRA) under BioProject accession PRJNA1445229. The 12S sequences generated for *Sarmarutilus rubilio* in this study were deposited in the GenBank database under accession numbers PX842949–PX842951.
