# Supplementary material for: Go With the Flow: Environmental Conditions Are Associated With Congruence Between eDNA Signals and Fish Communities in Small Lotic Ecosystems
Source: Ecol Evol. 2026 Nov 15;16(11):e74667. doi: 10.1002/ece3.74667 (PMC13770547; doi:10.1002/ece3.74667)
Supplement: Supplementary file 1 [file ECE3-16-e74667-s002.docx]

**Figure S1.** Boxplots of Bray-Curtis intra-site distances calculated using the number of eDNA reads (eDNA) and fish density (DI) or biomass (SC).

## Figure S2. Associations between proxies of eDNA and fish density and biomass. Plots depict the relationships (with 95% confidence intervals; shaded area) between square-root transformed eDNA reads and (A) fish abundance (DI) or (B) biomass (SC). Predictions are derived from the zero-inflated Gamma GLMMs presented in Table 2.
